# Nonocclusive mesenteric ischemia after percutaneous kyphoplasty: A case report

**DOI:** 10.1097/MD.0000000000039390

**Published:** 2024-08-23

**Authors:** Pengrui Wang, Meina Song, Xinxin Zhu, Weihong Ren, Haixiao Li, Sanli Cao, Shuhua Sun, Wei Pan, Shaohui Shi

**Affiliations:** a Department of Orthopedic Trauma, Aviation General Hospital, Beijing, PR China; b Department of Radiology, Aviation General Hospital, Beijing, PR China; c Department of General Surgery, Aviation General Hospital, Beijing, PR China; d Department of Pathology, Aviation General Hospital, Beijing, PR China.

**Keywords:** case report, nonocclusive mesenteric ischemia, percutaneous kyphoplasty, vertebral fracture

## Abstract

**Rationale:**

Percutaneous kyphoplasty (PKP) is a minimally invasive technique employed for treating vertebral compression fractures. Although PKP is simple and relatively safe, severe complications are possible. Here, we report a new, severe complication linked to this procedure, namely nonocclusive mesenteric ischemia (NOMI).

**Patient concerns:**

An 83-year-old female patient, previously in good health, fell backward, landing on her buttocks, and subsequently experienced persistent low-back pain that exacerbated during turning or sitting up.

**Diagnoses:**

Lumbar spine radiography revealed wedge deformity of the L1 vertebral body. Lumbar spine magnetic resonance imaging indicated a fresh compression fracture of the L1 vertebral body.

**Interventions:**

On the 2nd day following the trauma, the patient underwent PKP under local anesthesia. Anesthesia was satisfactory, and the procedure progressed smoothly.

**Outcomes:**

The patient experienced mild discomfort in the right abdomen within the 1st hour to 3 days postoperatively, mild abdominal distension on the 4th day, and sudden severe abdominal pain on the 5th day. Immediate abdominal computed tomography revealed ischemic changes in the ascending colon and hepatic flexure, accompanied by hepatic portal venous gas. An hour later, abdominal pain spontaneously subsided. Approximately 5 hours later, an enhanced abdominal computed tomography revealed no filling defects in the mesenteric vasculature, absence of luminal narrowing or occlusion, enhanced intestinal walls, and a notable improvement in hepatic portal venous gas. Considering NOMI and ischemia related to superior mesenteric artery spasm, vasodilator therapy (papaverine hydrochloride) was initiated, leading to favorable outcomes. On day 17, pathological examination of the hepatic flexure revealed moderate, acute, and chronic mucosal inflammation, along with interstitial fibrous tissue proliferation, providing clear evidence supporting ischemic changes. She was discharged on day 18 after a successful recovery.

**Lessons:**

The occurrence of NOMI after PKP is uncommon. Yet, once it happens, delayed diagnosis or misdiagnosis can lead to serious consequences such as intestinal necrosis and abdominal infection, even endangering the patient’s life. We currently lack experience in preventing this complication, but timely diagnosis and appropriate intervention are effective measures in treating such complications.

## 1. Introduction

Percutaneous kyphoplasty (PKP) is a standard approach for treating vertebral compression fractures by injecting polymethyl methacrylate into the affected vertebral body. The procedure aims to alleviate patient’s pain and achieve vertebral stability. With small incisions, simple operation, and a good safety profile, PKP is widely used. Complications associated with mesenteric ischemia in relation to this procedure are extremely rare, with only one reported case in the literature, which was linked to cement leakage.^[1]^ In October 2023, a patient with a fresh L1 vertebral body fracture was treated using PKP in our hospital. Postoperatively, the patient developed mesenteric ischemia without evidence of cement leakage. Nonocclusive mesenteric ischemia (NOMI) was diagnosed by our multidisciplinary team. This report combines the case report with the literature review to analyze the risk factors and preventive measures for such complications.

## 2. Case presentation

**Figure 1.**
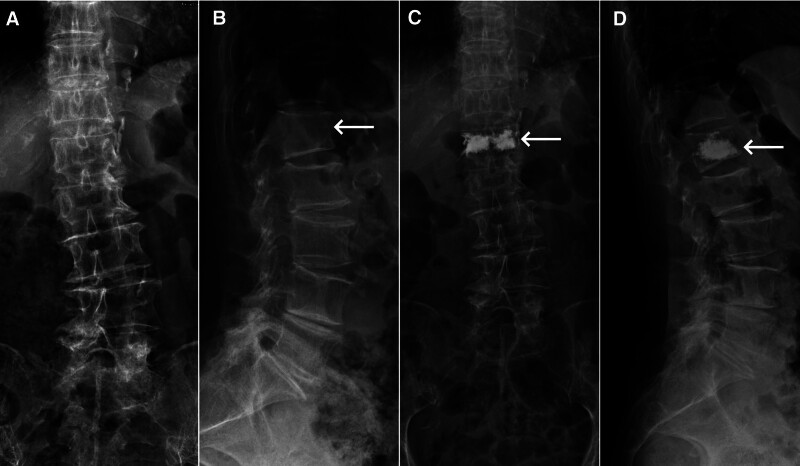
(A) Preoperative anteroposterior radiography showed lumbar degeneration with mild lateral curvature. (B) Preoperative lateral radiography revealed wedge-shaped deformation of the L1 vertebral body (arrow), showing anterior height loss without posterior rupture; no fracture fragments penetrating the spinal canal were observed. (C, D) Postoperative anteroposterior (C) and lateral (D) radiographies exhibited a mass-like high-density shadow (arrow) within the L1 vertebral body, with no free high-density shadows observed around the vertebral body.

**Figure 2.**
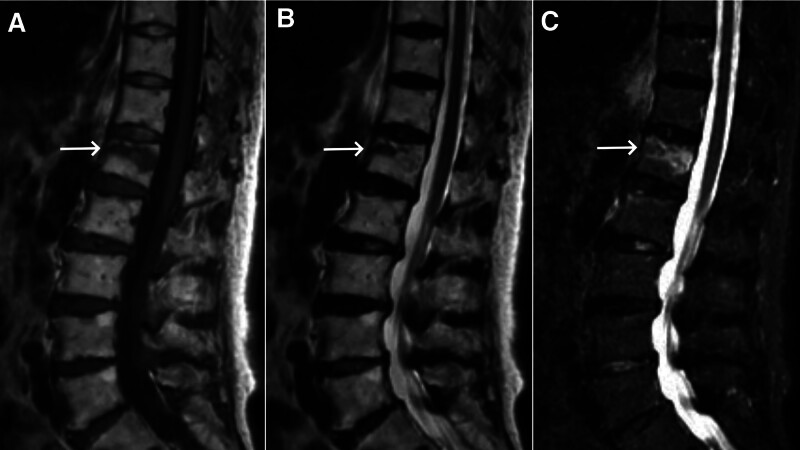
(A) Preoperative magnetic resonance imaging (MRI) in the T1-weighted phase revealed patchy, long T1 signal shadows (arrow) within the L1 vertebral body. (B) Preoperative MRI in the T2-weighted phase showed patchy, long T2 signal shadows (arrow) within the L1 vertebral body. (C) Preoperative MRI with fat suppression imaging showed patchy high signal (arrow) within the L1 vertebral body.

An 83-year-old female patient, previously in good health, fell backward, landing on her buttocks, and subsequently experienced persistent low-back pain that exacerbated during turning or sitting up. No pain or numbness was reported in the lower limbs. Physical examination revealed tenderness in the thoracolumbar region. Sensation and movement in both lower limbs were normal, with regular muscle tone. Bilateral knee reflexes were symmetrically elicited, and Babinski sign was absent on both feet. Lumbar spine radiography revealed mild lateral curvature of the lumbar spine (Fig. 1A) and wedge deformity of the L1 vertebral body (Fig. 1B). Lumbar spine magnetic resonance imaging (MRI) indicated a fresh compression fracture of the L1 vertebral body (Fig. 2A–C). On the 2nd day following the trauma, the patient underwent PKP under local anesthesia, positioned prone with the abdomen suspended, and guided by a G-arm image intensifier. Anesthesia was satisfactory, and the procedure progressed smoothly with 25 fluoroscopic shots. The total pronation time was 40 minutes, of which 20 minutes were spent on the surgery. The injected bone cement volume was 5 mL (2 mL on the left and 3 mL on the right). Postoperative radiography confirmed a satisfactory distribution of the cement within the L1 vertebral body, with no signs of leakage (Fig. 1C and D).

**Figure 3.**
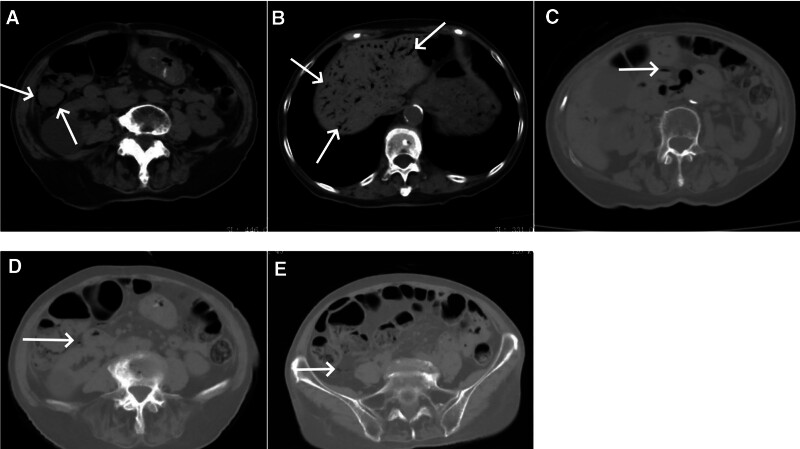
Abdominal computed tomography (CT) conducted during the onset of severe abdominal pain on the 5th day after operation. (A) Thickening of the colonic hepatic flexure wall with densely clustered small cystic lucencies (arrows) within the wall. (B) Abundance of branching lucencies (arrows) within the liver along the portal vein distribution, gradually tapering from the porta hepatis toward the periphery, presenting a “dead tree branch” appearance. (C) Presence of gas within the superior mesenteric vein (arrow). (D) Minimal gas accumulation in the right colonic vein (arrow). (E) Slight gas accumulation in the ileocolic vein (arrow).

The patient experienced occasional mild discomfort in the right abdomen from 1 hour to 3 days after PKP. No specific treatment was administered, and the patient was encouraged to ambulate with the assistance of a waist support. On the 4th day after PKP, the patient reported mild abdominal bloating, for which no specific treatment was provided. On the 5th day postoperatively, the patient suddenly developed severe abdominal pain (10/10 on visual analog scale), without fever, nausea, vomiting, diarrhea, or hematochezia. The patient was alert, and vital signs were stable. Abdominal palpation revealed softness but right abdominal tenderness without rebound tenderness or muscle tension. An urgent abdominal computed tomography (CT) scan revealed edema in the ascending colon and hepatic flexure, along with gas accumulation in the intestinal wall, mesenteric veins, and portal vein (Fig. 3A–E). Emergency blood tests indicated a white blood cell count of 7.79 × 10^9^/L with a neutrophil percentage of 78.3%. A consultation with the general surgery team was arranged. Following the consultation, the preliminary diagnosis was necrotizing colitis with signs of portal venous gas. The patient and her family were briefed on the possibility of rapid deterioration of the patient’s condition, accompanied by severe electrolyte imbalance, systemic inflammatory response syndrome, sepsis, and other related concerns. Urgent surgery, combined with effective antimicrobial therapy, was the preferred approach. An emergency laparotomy was planned for the patient, intending to perform resection of the necrotic colon and colostomy. After being apprized of the potential benefits and risks associated with the surgical intervention, both the patient and her family provided informed consent for the procedure, notwithstanding their concerns regarding the risks and complications.

**Figure 4.**
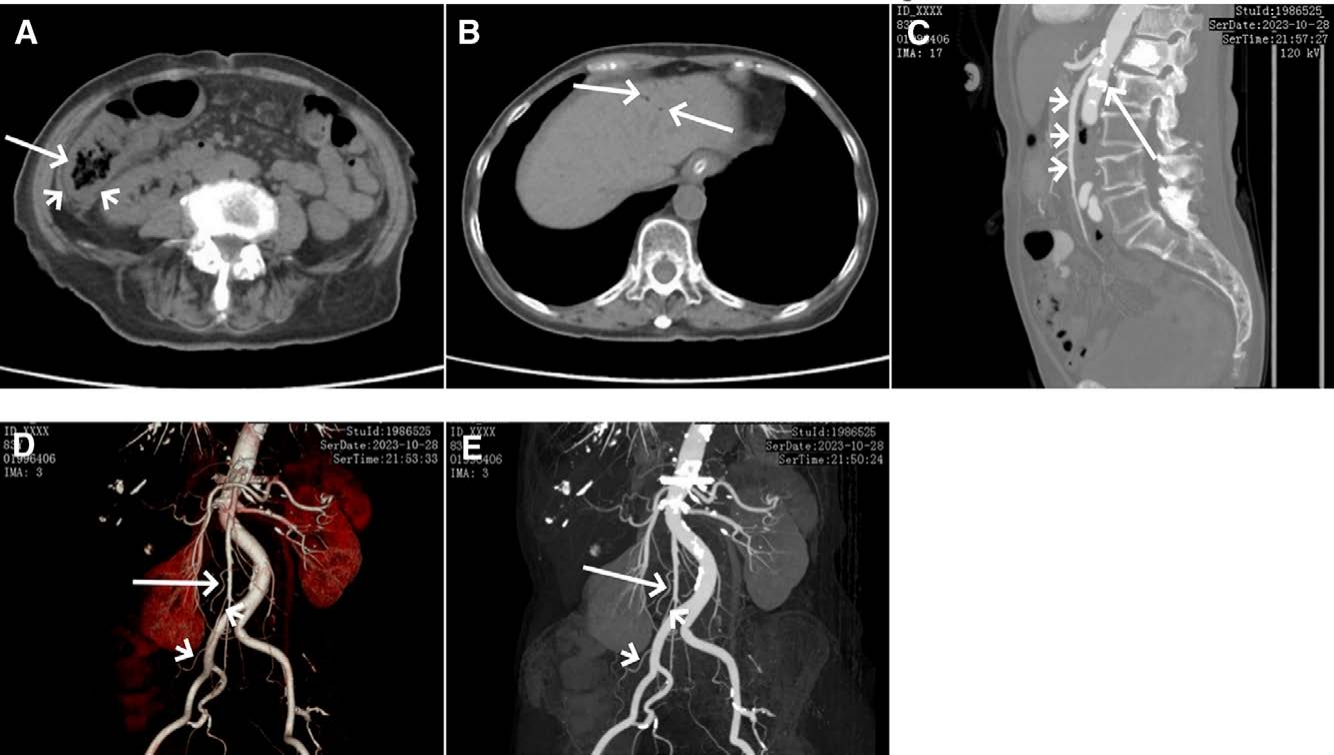
Enhanced abdominal computed tomography (CT) performed 5 hours after the sudden onset of severe abdominal pain on the 5th postoperative day. (A) Thickening of the colonic hepatic flexure wall (arrow) with disappearance of intramural gas and blurry infiltration of the surrounding fat spaces (arrowheads). (B) Substantial reduction in gas within the intrahepatic portal veins, with slight residual gas (arrows) in the left upper segment of the liver. (C) Multiple calcified plaques (arrow) visible in the abdominal aortic wall, with no filling defect in the SMA (arrowheads). (D, E) The natural course of the right colic artery (arrow) and the ileocolic artery (arrowheads) is shown, without filling defects, stenosis, or occlusion.

As part of the active preoperative preparation, the patient underwent fasting, skin preparation, gastrointestinal decompression, glycerin enema, and infection prevention. The Medical Affairs Department organized a multidisciplinary team for diagnosis and treatment. Approximately 1 hour later, the patient’s abdominal pain was spontaneously reduced, but dull pain (4/10 on visual analog scale) persisted. Physical examination remained consistent with previous findings. About 3 hours later, a follow-up blood test revealed white blood cell count of 8.99 × 10^9^/L, neutrophil percentage of 91.3%, procalcitonin level < 0.05 ng/mL, C-reactive protein level of 11 mg/L, and D-dimer level of 11.03 mg/L. Approximately 5 hours later, an enhanced abdominal CT showed increased thickening in the ascending colon and hepatic flexure, reduced bowel dilation, and significant absorption of gas in the intestinal wall, mesenteric veins, and portal vein (Fig. 4A and B). The mesenteric vessels showed no signs of filling defects, luminal narrowing, or occlusion. The superior mesenteric artery (SMA), right colic artery, and the ileocolic artery exhibit a normal course (Fig. 4C–E). The intestinal wall showed enhancement. The multidisciplinary consultation team (Medical Affairs, Gastroenterology, General Surgery, Radiology, Anesthesiology, Intensive Care Medicine, Cardiology, Neurology) provided the following opinions: hepatic portal venous gas (HPVG) was deemed a transient event, consistent with localized mucosal injury or necrosis with gas extravasation. As the patient had no preceding symptoms of diarrhea or hematochezia, necrotizing colitis was not considered; at the time, there was significant edema with exudation in the ascending colon and hepatic flexure, corresponding to the distribution area supplied by the right colic artery and the ileocolic artery. Enhanced CT showed the entire course of the SMA, right colic artery, and ileocolic artery, with no signs of weakened or absent bowel wall enhancement. We believe that factors such as vascular spasm could not be ruled out; Although the patient experienced sudden and severe abdominal pain, it resolved spontaneously, and there was no worsening of vital signs. Although there was an elevation in white blood cell count and neutrophil percentage, the white blood cell count remained within the normal range. Procalcitonin levels were within the normal range, and C-reactive protein concentration showed a mild elevation. Imaging did not show any signs of bowel perforation or gastrointestinal bleeding. At that time, vital signs were stable, the general condition was good, and observation of the condition was deemed appropriate. Additionally, due to the unclear scope of intestinal lesions, it was considered that proceeding with surgery hastily might entail the risk of multiple surgeries. Therefore, surgical intervention was not recommended at that time; in terms of treatment, papaverine was administered for vasodilation. Continuous measures such as fasting, gastrointestinal decompression, and infection prevention were maintained. Somatostatin was not employed, so as to avoid worsening intestinal ischemia. Thrombolytic therapy and heparin anticoagulation were not considered because of insufficient evidence. Clinical evidence suggested that administering 60 mg papaverine hydrochloride, diluted in 250 mL of saline solution and slowly infused intravenously, could further alleviate abdominal pain (2/10 on visual analog scale) Subsequently, a daily dosage of 120 mg papaverine hydrochloride, administered intravenously in 2 divided doses, was given. Mild abdominal pain (2/10 on visual analog scale) persisted for 6 to 8 days after operation. Medication was discontinued after the abdominal pain subsided on the 9th day after operation.

**Figure 5.**
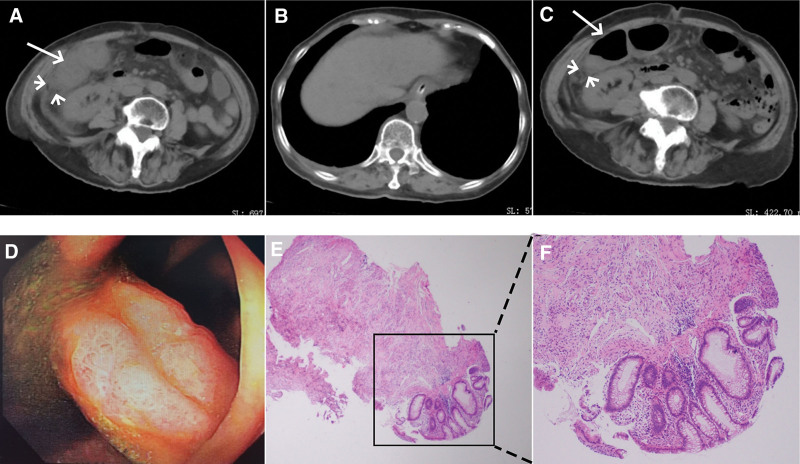
(A) On the 7th postoperative day, there was increased thickening and edema in the colonic hepatic flexure wall (arrow), accompanied by an enlarged range of surrounding fat infiltration (arrowheads). (B) On the 7th postoperative day, complete absorption of gas within the portal vein was evident. (C) By the 9th postoperative day, there was an observable alleviation of the thickening and edema in the colonic hepatic flexure wall (arrow), with a reduced range of surrounding fat infiltration (arrowheads). (D) Fifteen days postoperatively, colonoscopy revealed mucosal edema in the colonic hepatic flexure, with glandular dilation on the mucosal surface. Focal areas showed the formation of white scars. (E, F) Seventeen days postoperatively, histopathological examination (HE staining, 4×) (E) and (HE staining, 10×) (F) revealed moderate, acute, and chronic inflammatory cell infiltration in the mucosal and submucosal layers, accompanied by increased fibrous tissue proliferation.

On the 7th postoperative day, a follow-up abdominal CT revealed an increased thickening and edema of the colonic hepatic flexure wall, with an expanded range of surrounding fat infiltration (Fig. 5A). Complete absorption of gas was observed in the intrahepatic portal veins (Fig. 5B). On the 9th postoperative day, there was a relief in the thickening and edema of the colonic hepatic flexure wall, with a reduction in the extent of surrounding fat infiltration (Fig. 5C). On the 15th postoperative day, electronic colonoscopy revealed mucosal edema in the colonic hepatic flexure, glandular ductal dilation on the mucosal surface, focal white scar formation, and luminal narrowing (Fig. 5D). Biopsy samples were obtained from significant lesions for pathological examination. No strictures, edema, ulcerations, or erosions were found in the remaining rectum. Pathological analysis on the 17th day indicated moderate, acute, and chronic inflammation of the mucosa, along with increased interstitial fibrous tissue (Fig. 5E and F), providing a clear confirmation of the diagnosis of ischemic colitis. The patient recovered and was discharged on the 18th postoperative day. Telephone follow-up was conducted at 1, 2, and 3 months after discharge. The patient reported no abdominal discomfort and no pain in the lumbar region. She demonstrated self-care ability and unrestricted mobility in daily activities.

## 3. Discussion

Vertebral cement augmentation procedures, such as PKP or percutaneous vertebroplasty (PVP), are commonly employed to alleviate pain in the vertebral column, often associated with fractures resulting from traumatic injuries, osteoporosis, or metastatic lesions.^[2–4]^ Complications related to arterial ischemia are relatively uncommon in vertebral cement augmentation procedures. Current reports primarily implicate the lumbar artery,^[5]^ anterior spinal artery,^[6,7]^ mesenteric artery,^[1]^ and intercostal segmental artery.^[8]^ These cases all involve mechanical stenosis or obstruction, and hitherto, there have been no reported instances of nonarterial stenosis or obstruction. Recently, a patient with an L1 vertebral body fracture was treated at our orthopedic medical center treated using PKP. Following the procedure, the patient developed mesenteric ischemia. Investigation ruled out factors such as cement leakage, direct injury, bone cement implantation syndrome (BCIS), mesenteric vascular thrombosis, and embolism. There was a high suspicion of NOMI, with consideration of mesenteric artery spasm. Treatment with papaverine hydrochloride for spasm relief proved effective, leading to a successful recovery and discharge.

Postoperative lumbar spine radiographs and abdominal CT scans showed a well-distributed bone cement within the vertebral bodies, and no signs of leakage beyond the vertebrae were found. This eliminated concerns regarding cement leakage.

The intraoperative G-arm-guided puncture was smooth, without penetrating beyond the pedicle or vertebral body, ruling out the possibility of a direct injury.

During the application of bone cement, BCIS can occur, manifesting as hypoxia, systemic hypotension, pulmonary hypertension, arrhythmias, loss of consciousness, and eventual cardiac arrest.^[9]^ BCIS is more common in cemented joint replacement surgeries^[10,11]^ and less frequent in procedures such as PKP and PVP.^[12,13]^ The etiology and pathophysiology of BCIS remain unclear. Early theories emphasized the toxic effects of circulating monomers from methyl methacrylate (MMA). Although MMA can permeate into the bloodstream, causing peripheral vasodilation and exhibiting cardiac, pulmonary, vascular,^[14]^ and cellular toxicity,^[15]^ this theory is losing support due to the high concentrations of MMA required to induce hemodynamic changes in humans.^[16,17]^ Recent theories propose an embolus-mediated model for BCIS, but not all documented BCIS occurrences align with this theory alone, requiring further research in this area.^[18]^ In this case, the patient showed no abnormal changes in hemodynamics or respiratory function throughout the entire intraoperative and postoperative period. Consequently, concerns related to BCIS and MMA monomer toxicity were not considered.

Occlusive mesenteric arterial ischemia typically presents as either mesenteric arterial embolism or thrombosis, with the SMA often being the affected vessel. A major cause of mesenteric vascular embolism is atrial fibrillation.^[19,20]^ Acute SMA embolism is characterized by severe abdominal pain, potentially disproportionate to clinical signs, along with intense gastrointestinal symptoms such as nausea, vomiting, or diarrhea.^[21,22]^ Arterial thrombosis commonly results from severe atherosclerosis.^[20,23]^ Patients with arterial thrombosis often show signs of chronic mesenteric arterial ischemia, presenting as postprandial abdominal pain leading to food avoidance, gradual weight loss, and symptoms of chronic malabsorption, including chronic diarrhea.^[24,25]^ In cases where thrombosis causes acute vascular obstruction, clinical presentation resembles that of acute artery embolism. Enhanced CT is a valuable diagnostic tool, offering high specificity, sensitivity, and noninvasiveness.^[25]^ CT-specific findings for acute mesenteric ischemia (AMI) include thrombotic emboli in mesenteric arteries, narrowing or occlusion of mesenteric arteries, and decreased or absent enhancement of the intestinal wall. D-dimer testing for AMI is highly sensitive but lacks specificity. In this case, the patient’s characteristics were as follows: the patient was an elderly female with no history of cardiovascular disease, no signs of atrial fibrillation, and no symptoms such as postprandial abdominal pain or chronic diarrhea; throughout the course of this illness, there were no episodes of vomiting, diarrhea, or severe abdominal signs. Vital signs remained stable; although the patient had no history of cardiovascular disease, the abdominal CT scan (Fig. 4C) revealed quite severe aortic arteriosclerosis in the abdominal aortic wall and the proximal part of the SMA. Thus, acute SMA embolism caused by mesenteric arterial embolism or thrombosis could not be readily ruled out. However, approximately 1 hour after the acute onset of symptoms, the abdominal pain spontaneously subsided. Furthermore, enhanced abdominal CT showed the entire course of the SMA, right colic artery, and ileocolic artery, with a natural course and no evidence of filling defects, narrowing, or occlusion; the significantly elevated D-dimer level of 11.03 mg/L, well above the normal upper limit, was attributed to stress response and was not considered indicative of mesenteric thrombosis. The patient received no thrombolysis or anticoagulant therapy throughout the illness, with no impact on prognosis. Based on the patient’s history of past illness, history of present illness, and diagnostic process, we did not consider mesenteric arterial embolism or thrombosis.

“Holism” is a guiding principle that primarily encompasses 2 aspects: first, attempting to explain multiple system or organ pathologies with a single etiology; second, striving to interpret various clinical presentations with a single disease entity. Only when a patient’s clinical manifestations cannot be adequately explained by a single disease should other possibilities be considered. This principle is particularly highlighted in diagnosing challenging diseases. Adhering to the principle of “Holism,” we found that NOMI provides a more reasonable explanation for the entire development process of intestinal ischemia in this patient. NOMI is defined as a subtype of mesenteric ischemia without thrombotic occlusion of blood vessels.^[26]^ NOMI is usually regarded as a spasm of the splanchnic vessels, especially the SMA.^[20]^ Early NOMI symptoms are often atypical, presenting as nonspecific discomfort such as abdominal bloating.^[27]^ Radiological features on enhanced CT include the absence of mechanical intestinal obstruction, no proximal embolism or thrombosis in the mesenteric arteries, and minimal enhancement of the intestinal wall. The core principle in managing NOMI is addressing underlying causative factors.^[25]^ Administration of vasodilators such as papaverine significantly expands peripheral blood vessels, increases blood flow, and markedly reduces in-hospital mortality and prevalence of abdominal surgery in patients with NOMI.^[28,29]^ Initial ischemic damage is usually confined to the mucosa and is reversible if spasmogenic factors are eliminated.^[30]^ Failure to remove these factors can lead to transmural necrosis of the intestinal wall, which is irreversible even after eliminating the causative factors. In a recent study, the “time from CT to injecting vasodilator” has emerged as the sole survival factor for NOMI patients.^[31]^ Early symptoms in our case were also atypical, namely mild discomfort in the abdomen that occurred 1 hour to 3 days postoperatively, mild abdominal distension on day 4 postoperatively, and sudden abdominal pain on day 5 postoperatively. Enhanced CT revealed no signs of mechanical intestinal obstruction, and there was no evidence of embolism or thrombosis in the mesenteric arteries. The patient’s enhanced CT did not exhibit the typical signs of reduced intestinal wall enhancement or non-enhancement, possibly due to the timing of the CT scan. The acute abdominal pain (AAP) spontaneously subsided approximately 1 hour after its onset, while the abdominal CT was completed around 5 hours later, potentially after the spontaneous relief of mesenteric artery spasm. Our hospital’s multidisciplinary team collectively considered this case as NOMI, and prompt intervention with papaverine hydrochloride yielded an excellent result. Here, it should be noted that NOMI is usually regarded as a spasm of the splanchnic vessels, especially the SMA, which simplifies a more complex multifactorial process. NOMI is generally associated with impaired SMA flow following systemic hypoperfusion related to a low-flow state. Common causes involve Low Cardiac Output Syndrome, episodes of hypotension, and vasopressor therapy (e.g., norepinephrine). However, during the entire illness, including the patient’s sudden and severe abdominal pain episode, vital signs remained stable without evidence of hypotension, hypoxia, narrow pulse pressure, altered consciousness, cold extremities, peripheral cyanosis, weak pulses, slow capillary refill time, oliguria, or anuria. In this case, we did not observe typical clinical manifestations of Low Cardiac Output Syndrome or episodes of hypotension before or after surgery. Furthermore, vasopressor therapy (e.g., norepinephrine) was not utilized.

High lactate concentrations are widely recognized as prominent biomarkers for detecting mesenteric ischemia. Several studies have documented the diagnostic value of lactate in AMI.^[22,32,33]^ However, some studies have reported that the sensitivity of lactate is inadequate.^[26,34,35]^ Matsumoto et al^[34]^ demonstrated that lactate has a sensitivity of 60% (area under the curve, 0.646) in diagnosing NOMI. Hong et al^[35]^ reported a lactate sensitivity of 39% (area under the curve, 0.510). Furthermore, some researchers have observed that serum lactate serves as a prognostic indicator rather than a diagnostic biomarker for AMI, and a normal serum lactate level does not rule out the diagnosis.^[20]^ In this case, although there were fluctuations in lactate levels, the upward trend was not significant, and the levels remained within normal limits. Lactate levels peaked on the 1st day after the onset of AAP and returned to preoperative levels by the 4th day (Table 1). This may be related to the low sensitivity of lactate levels reported in some literature.^[26,34,35]^ Additionally, the patient’s AAP spontaneously ameliorated approximately 1 hour later, which may have interrupted the rising trend of lactate levels. Furthermore, the timely administration of papaverine hydrochloride to alleviate the spasms could also have influenced changes in lactate levels. Overall, the patient remained clinically stable with a favorable prognosis. Drawing from the literature,^[20]^ a tentative hypothesis posits that this case supports the correlation between low lactate levels and a favorable prognosis.

**Table 1:** Lactate levels during complications (normal range 0.5–2.2 mmol/L).

	Preoperative	Within 1 h After AAP	1 d After AAP	2 d After AAP	3 d After AAP	4 d After AAP
Lactate (mmol/L)	0.7	0.8	1.1	1.0	1.0	0.7

AAP = acute abdominal pain.

NOMI is commonly associated with the blood supply region of the SMA.^[20]^ In this case, the patient exhibited ischemic changes in the ascending colon and hepatic flexure following L1 vertebral body PKP, aligning with the distribution territory of the right colic artery and the ileocolic artery. Anatomically, the SMA gives rise to the right colic artery and the ileocolic artery.^[36]^ We postulate that the ischemia primarily originates from the SMA, which coincidentally arises at the level of the L1 vertebra from the anterior wall of the abdominal aorta. The precise mechanism by which bone cement affects the blood supply of the SMA remains unclear. Considering the patient’s underlying vascular condition and the time gap between the onset of NOMI and the use of bone cement, it is likely that NOMI may be associated with a preexisting stenosis of the SMA. However, the use of bone cement, while it may not be the major determinants, could also potentially be a contributing factor, leading to the delayed onset of impaired SMA flow. Additionally, the prone position could also be a contributing factor, potentially causing spasms in an atherosclerotic plaque.

Early detection and intervention carry significant therapeutic importance but pose challenges.^[37]^ In patients with vertebral fractures, the disease onset can lead to retroperitoneal hematoma, resulting in gastrointestinal dysfunction and discomfort such as abdominal distension. Post-vertebral augmentation procedures may also predispose patients to complications such as abdominal distension. As abdominal distension is relatively nonspecific and lacks immediate life-threatening implications, it can be easily overlooked during treatment. Therefore, timely and accurate identification of the nature and progression trends of postoperative abdominal distension, along with the development of corresponding intervention measures, is paramount. Treatment under a multidisciplinary team significantly reduces the patient’s risk of intestinal resection and mortality.^[25,38]^ Radiologists play a crucial role in early diagnosis before irreversible bowel necrosis occurs. Correct intervention holds absolute therapeutic significance.^[39,40]^ Early vascular reconstruction and intensive care contribute to reducing mortality rates.^[41]^ This emphasizes the importance for admitting physicians to conduct a meticulous physical examination, integrate the patient’s medical history, and make a comprehensive analysis to assess and judge the condition.

In summary, the development of NOMI following PKP is a rare complication. The exact mechanism by which bone cement affects the blood supply of the SMA remains unclear. Hitherto, there has been limited experience in preventing this complication. However, timely diagnosis and appropriate intervention are effective measures for managing this complication.

## Acknowledgments

The authors thank the patient and her family for allowing this paper to be published. We thank the Hospital Medical Affairs Department and the multidisciplinary team for their support. We thank LetPub (www.letpub.com) for its linguistic assistance during the preparation of this manuscript.

## Author contributions

**Conceptualization:** Pengrui Wang, Meina Song, Xinxin Zhu, Haixiao Li, Sanli Cao, Shuhua Sun, Wei Pan, Shaohui Shi.

**Data curation:** Pengrui Wang, Meina Song, Xinxin Zhu, Weihong Ren, Sanli Cao, Shuhua Sun, Wei Pan, Shaohui Shi.

**Investigation:** Pengrui Wang, Meina Song, Xinxin Zhu, Weihong Ren, Haixiao Li.

**Methodology:** Pengrui Wang.

**Supervision:** Xinxin Zhu, Weihong Ren, Shaohui Shi.

**Validation:** Pengrui Wang, Haixiao Li, Shaohui Shi.

**Visualization:** Pengrui Wang, Meina Song, Weihong Ren, Sanli Cao, Shuhua Sun, Wei Pan, Shaohui Shi.

**Writing – original draft:** Pengrui Wang.

**Writing – review & editing:** Shaohui Shi.
